# On the move: Influence of animal movements on count error during drone surveys

**DOI:** 10.1002/ece3.70287

**Published:** 2024-09-29

**Authors:** Emma A. Schultz, Natasha Ellison‐Neary, Melanie R. Boudreau, Garrett M. Street, Landon R. Jones, Kristine O. Evans, Raymond B. Iglay

**Affiliations:** ^1^ Department of Wildlife, Fisheries and Aquaculture Mississippi State University Mississippi State Mississippi USA; ^2^ Quantitative Ecology and Spatial Technologies Lab Mississippi State University Mississippi State Mississippi USA

**Keywords:** agent‐based model, count bias, remotely piloted aircraft system, survey error, unmanned aerial vehicle, unoccupied aircraft system

## Abstract

The use of remote sensing to monitor animal populations has greatly expanded during the last decade. Drones (i.e., Unoccupied Aircraft Systems or UAS) provide a cost‐ and time‐efficient remote sensing option to survey animals in various landscapes and sampling conditions. However, drone‐based surveys may also introduce counting errors, especially when monitoring mobile animals. Using an agent‐based model simulation approach, we evaluated the error associated with counting a single animal across various drone flight patterns under three animal movement strategies (random, directional persistence, and biased toward a resource) among five animal speeds (2, 4, 6, 8, 10 m/s). Flight patterns represented increasing spatial independence (ranging from lawnmower pattern with image overlap to systematic point counts). Simulation results indicated that flight pattern was the most important variable influencing count accuracy, followed by the type of animal movement pattern, and then animal speed. A  awnmower pattern with 0% overlap produced the most accurate count of a solitary, moving animal on a landscape (average count of 1.1 ± 0.6) regardless of the animal's movement pattern and speed. Image overlap flight patterns were more likely to result in multiple counts even when accounting for mosaicking. Based on our simulations, we recommend using a lawnmower pattern with 0% image overlap to minimize error and augment drone efficacy for animal surveys. Our work highlights the importance of understanding interactions between animal movements and drone survey design on count accuracy to inform the development of broad applications among diverse species and ecosystems.

## Graphical abstract

Drone flight pattern most influenced the count accuracy of a mobile animal. Large image overlaps frequently produced multiple counts of a moving animal. We recommend reducing overlap to increase the accuracy of drone surveys of mobile animals.

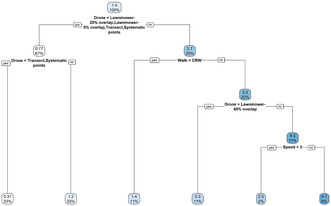

## 1 INTRODUCTION

Drones (i.e., unoccupied aircraft systems or UAS) are increasingly being used for myriad ecological applications, including direct animal observation (Hodgson et al., 2018; Koh & Wich, 2012; Vermeulen et al., 2013), vegetation evaluation (Olsoy et al., 2018, 2020), and nest observation (Lachman et al., 2020; Lyons et al., 2019). Benefits associated with using drones in animal monitoring, compared to traditional animal survey techniques, include less time and effort in the field (McMahon et al., 2022), reduced animal disturbance compared to ground surveys (Barr et al., 2020; Krause et al., 2021), and greater survey accuracy (Hodgson et al., 2018; Jones et al., 2020). Additionally, drones can be launched over areas inaccessible for ground surveys (Junda et al., 2015; Wang et al., 2019), provide a safer alternative for ecologists compared with occupied aircraft (Christie et al., 2016; Hartmann et al., 2021; Sasse, 2003), and enable creation of digital repositories of high‐resolution imagery from use of advanced sensor technologies (Samiappan et al., 2024; Wang et al., 2019). Drone use in animal monitoring continues to increase (Linchant et al., 2015), a trend that is exemplified by the recent annual publication rate of articles investigating animal surveys using drones during the past decade (Chabot, 2018; Elmore et al., 2023). However, drone surveys have limitations compared with traditional methods, including relatively short battery lives (Linchant et al., 2015), large post‐processing time requirements for images (Barbedo & Vieira Koenigkan, 2018), and line‐of‐sight restrictions (Chabot & Bird, 2015; Duffy et al., 2018). Additionally, drones may lead to behavioral changes or disturb animals of interest (Headland et al., 2021; Wilson et al., 2023), which may lead to inaccurate survey counts (Augustine & Burchfield, 2022) and can depend on a variety of factors (Mo & Bonatakis, 2021).

**FIGURE 1:**
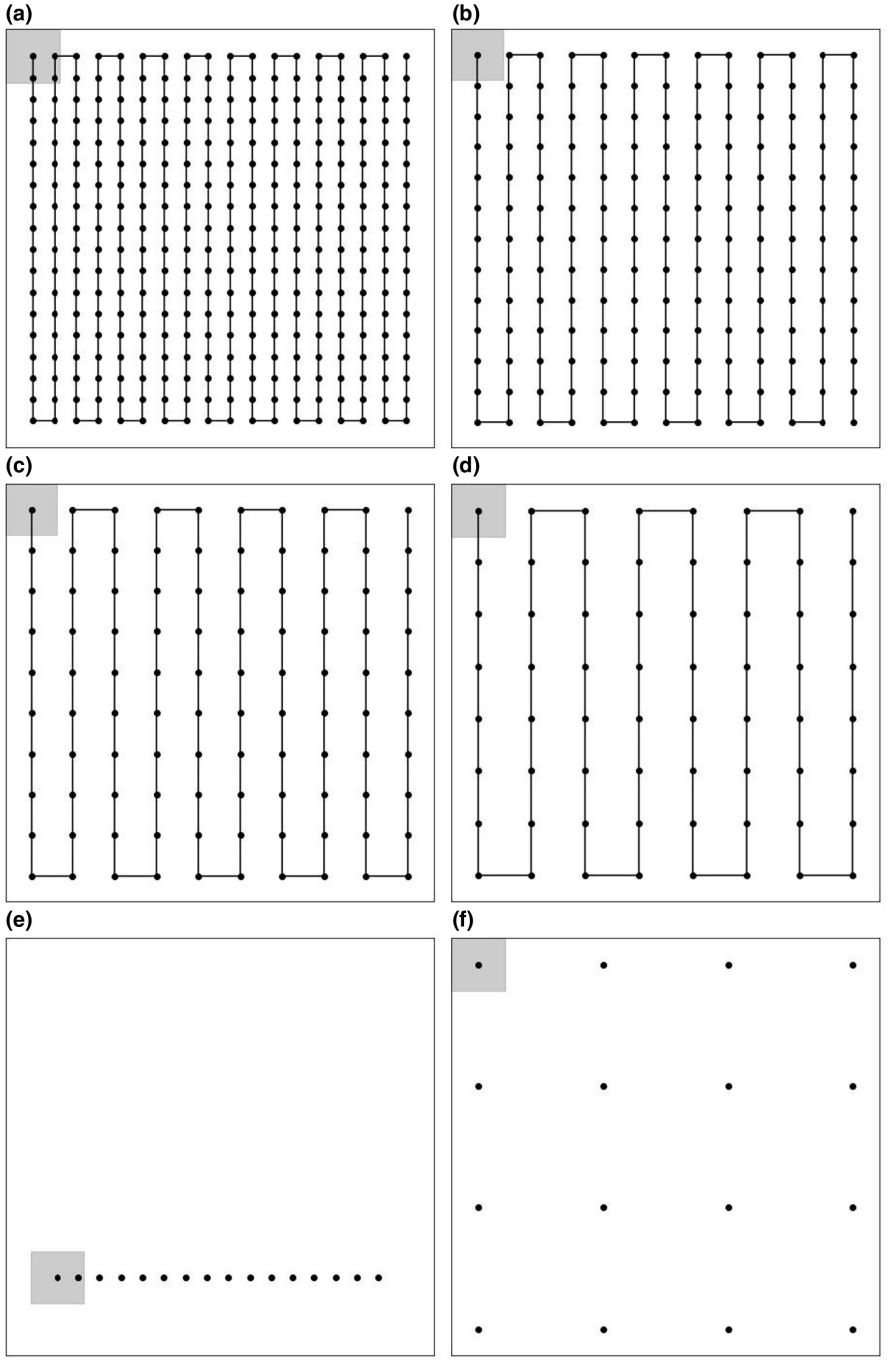
Drone flight patterns simulated over a theoretical landscape: (a) lawnmower with 60% overlap, (b) lawnmower with 40% overlap, (c) lawnmower with 20% overlap, (d) lawnmower with 0% overlap, (e) belt transect, (f) systematic points. Gray boxes denote viewing window of the first image taken during each survey representing a 60 × 60 m area.

Numerous survey methods are used in conservation science for population assessments and vary based on species of interest, landscape size and characteristics, as well as survey objectives (Silvy, 2020). Typical drone survey methods sample an area with a lawnmower (i.e., back and forth) pattern (Elmore et al., 2023). Belt transects are less common in drone surveys, and point counts, a common technique for ground surveys, could be adapted to drone surveys using programmed flight patterns (Silvy, 2020). Lawnmower patterns in drone surveys typically include 60%–80% frontal and side overlapping of adjacent images (Figure 1a–d; Ezat et al., 2018; Lyons et al., 2019; Aubert et al., 2021). While overlapping images are necessary for mapping orthorectified landscapes (Koh & Wich, 2012), image overlap for animal monitoring can increase sampling bias due to risk of repeatedly counting individuals (Brack et al., 2018; Lenzi et al., 2023). Yet, common default flight settings among commercially available drone software use overlapping lawnmower flight patterns (Frazier & Singh, 2021; Harris et al., 2019), an approach that may not support accurate surveys.

Animal movements have the potential to influence counting accuracy in drone surveys through omission of individuals or multiple counts often caused by the same animal(s) occurring in several overlapping images (Brack et al., 2018). Lenzi et al. (2023) mentioned “ghost” animals produced when overlapping drone images were mosaicked. These were individuals that moved during subsequent image capture, creating blurred or transparent animals on the final mosaicked photograph, leading to possible erroneous counts. However, even when transect and image overlaps do not occur, multiple counts of mobile animals in drone surveys can happen (Witczuk et al., 2018). The distance traveled by animals within a given period depends on many factors, including life history needs and a variety of abiotic (e.g., seasonal resources) and biotic (e.g., conspecific competition) influences (Nathan et al., 2008). For example, breeding colonies of nesting shorebirds often remain on their nests (i.e., fixed locations) for long periods of time during breeding seasons (Hodgson et al., 2016; Jones et al., 2020). In contrast, an adult cheetah has been recorded at a running speed of up to 29 m/s (Sharp, 1997). Animals are known to exhibit changes in activity period throughout the day, with white‐tailed deer, black bears (Lewis & Rachlow, 2011), and wolves (Merrill & Mech, 2003) all moving more frequently during crepuscular periods. Thus, variation in animal movement patterns and speeds depend on the species ecology and current environment.

Movement models can be used to depict various animal movement patterns along a spectrum of speeds with (1) random walks representing animals dispersing randomly on the landscape, (2) correlated random walks depicting animals moving with directional persistence, mimicking something analogous to migration, and (3) biased random walks depicting animal home ranging behavior in some cases (Codling et al., 2008). These movement models challenge the common assumption among traditional survey methods of animals being detected in their original position (i.e., no movement) and can be applied to understand the influence of animal movement on drone‐based survey count error. Only one study, to our knowledge, has quantified error and highlighted the importance of estimating detection probability for drone flight patterns when monitoring a mobile animal (Hodgson et al., 2017), the humpback whale, but their study has limited application to terrestrial systems.

Simulations represent an alternative and powerful approach to evaluate how animal movements can affect drone surveys. Simulations have been employed to investigate how various drone survey speeds and altitudes influence abundance and occupancy estimates (Baxter & Hamilton, 2018). The virtual environment can also provide insights not possible in real‐world settings due to field inconsistency and other potential confounding variables (e.g., image processing, observer biases, and varying detection rates). Agent‐based modeling (ABM; also referred to as individual‐based modeling) uses iterative computer simulations to incorporate real‐world parameters in a controlled environment, modeling scenarios that can address targeted research questions (Chudzinska et al., 2021; Hoegh et al., 2021). Here, we used an ABM simulation approach (Grimm et al., 2020) to (1) quantify error rates among six drone flight patterns and three common animal movement patterns at five different speeds and (2) provide suggestions for optimal drone flight patterns that minimize error associated with animal movement. Our ABM simulation approach permitted a robust examination of the potential influence of animal movements and drone flight patterns on survey count errors that would otherwise be difficult to replicate in field experiments. We predicted lawnmower flight patterns with overlapping images would overestimate true counts due to counting the same individual multiple times. We also predicted that subsampling methods such as belt transects and multiple single images (i.e., systematic point counts) would underestimate true counts due to a greater probability of omitting the moving animal. Finally, we predicted that an increase in animal speed and persistence in the directional movement of the animal would lead to overestimation as the animal could cross multiple images.

## 2 MATERIALS AND METHODS

### 2.1 Drone parameters

We examined the potential error among drone flight patterns and animal movement models (speed and movement pattern) using ABM simulations created in Python 3.9 (van Rossum & Drake, 2009). To realistically approximate methodologies that conservation practitioners currently employ, the simulated drone sensor was programmed to approximate specifications of a 20‐megapixel camera with a focal length of 6.8 mm and field of view of approximately 67 degrees. Flights were modeled at 61 m above ground level, representing one of several typical altitudes for animal monitoring using a multi‐copter drone (McEvoy et al., 2016; Wang et al., 2019) which has been shown to have zero or minimal behavioral impacts to several animal species (Barr et al., 2020; Krause et al., 2021). This altitude and sensor combination produced a 1.28 cm ground sample distance and captured a ~50 m × 65 m ground footprint for each image. For simplicity, we adjusted the ground viewing window in simulations to a 60 × 60 m area, with grid cells in our simulation measuring 4 m by 4 m in dimension. The drone speed was simulated at 10 m/s to approximate a realistic platform speed for image capture and sharpness. To approximate real‐world drone battery capabilities and line‐of‐sight considerations, surveys did not exceed a 30 min flight time (Raoult et al., 2020).

### 2.2 Landscape and drone flight patterns

**FIGURE 2:**
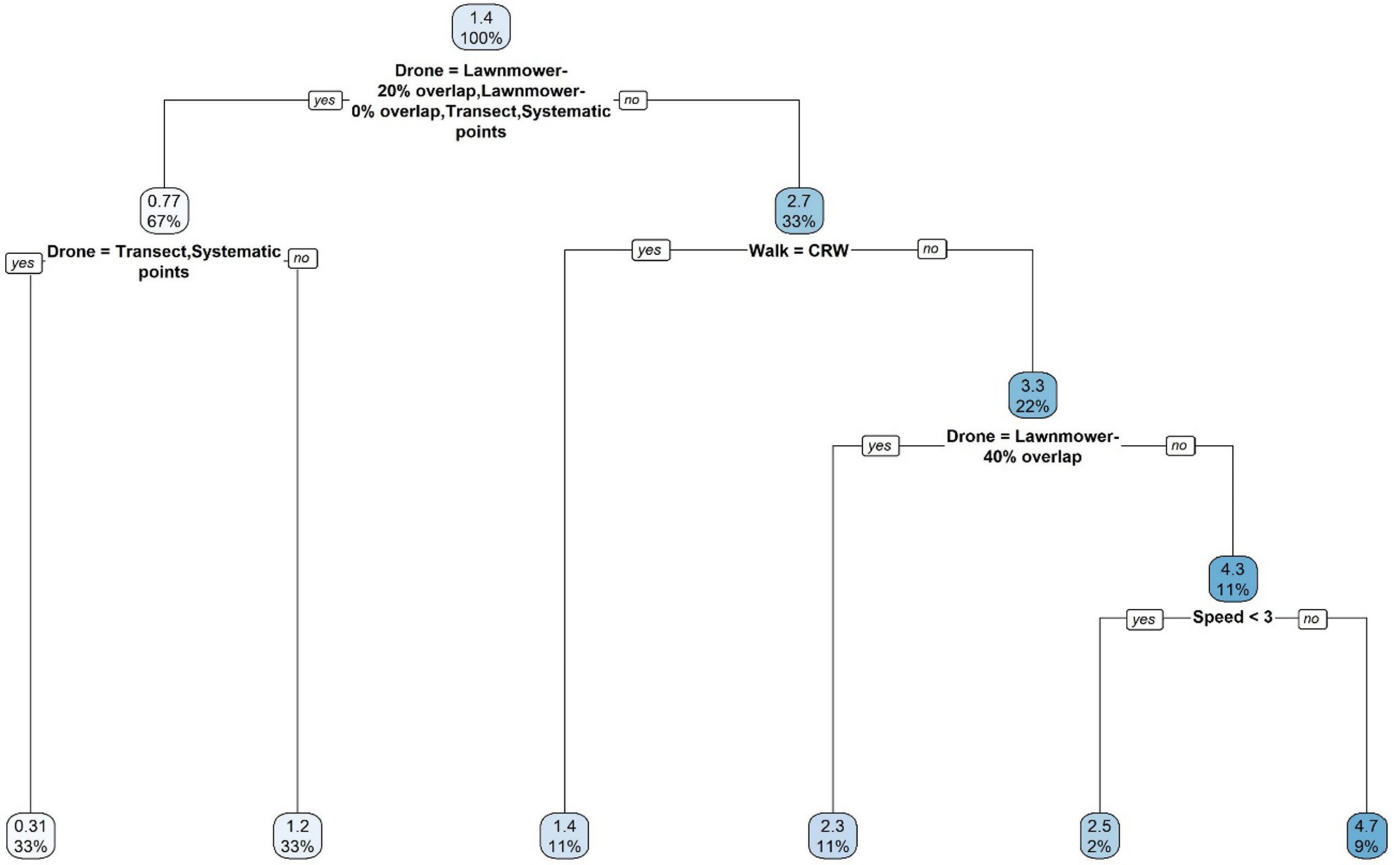
Regression tree analysis of an agent‐based model simulating various drone flight patterns across a landscape with raw count as the response variable. Splits indicate the importance of each predictor variable. At each node, splits to the left indicate “yes” and the right “no” based on the predictor variables listed. The numbers in each node represent the mean of the raw counts and the percentage of the total number of observations that fall within.

**FIGURE 3:**
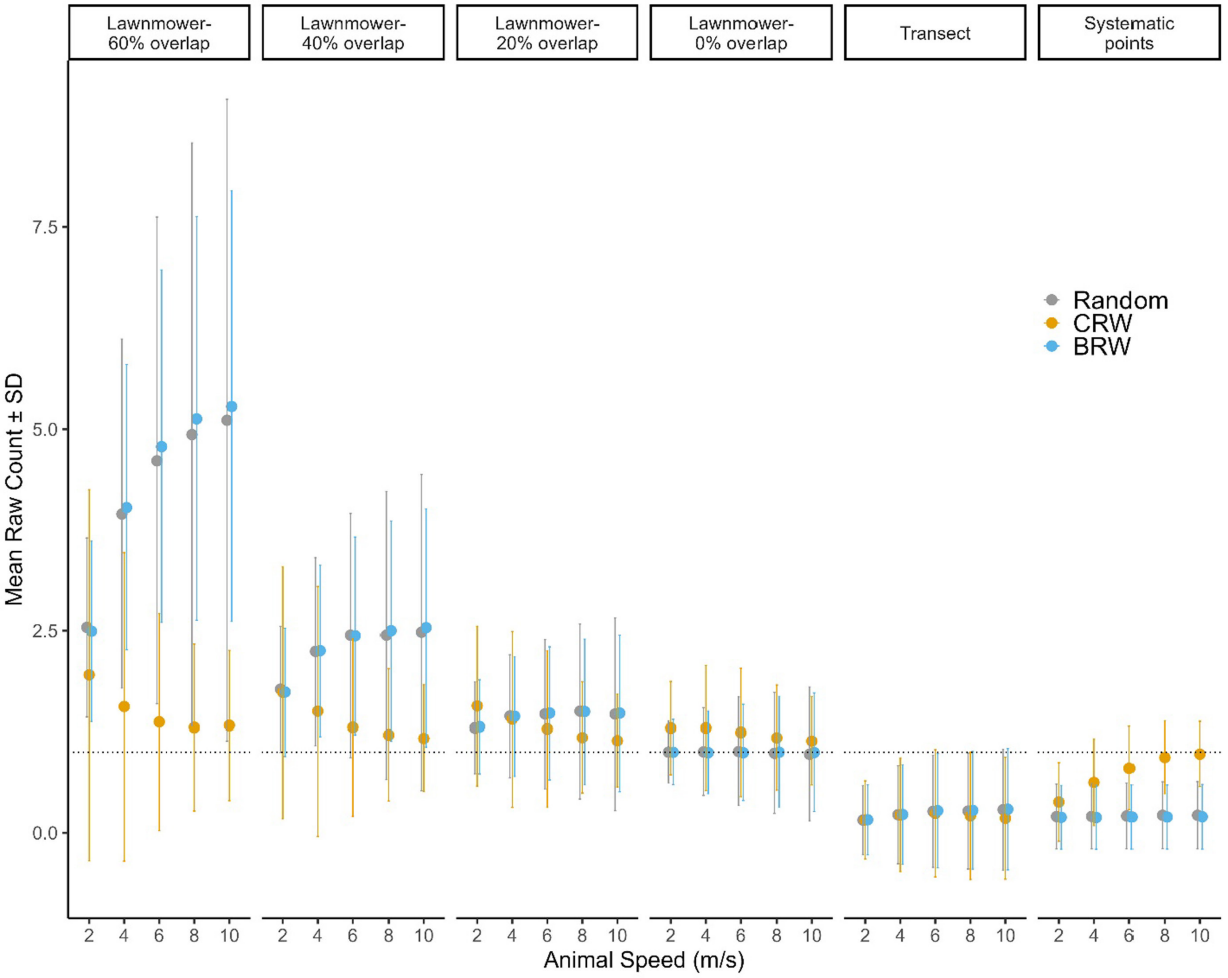
Mean and standard deviation for raw counts from 10,000 replicates of an agent‐based model simulating various drone flight patterns across a landscape. Dotted lines show a count of 1, which represents the one animal placed on the landscape. Animal movement patterns include random (no directional persistence), CRW, correlated random walk, BRW, biased random walk.

**FIGURE 4:**
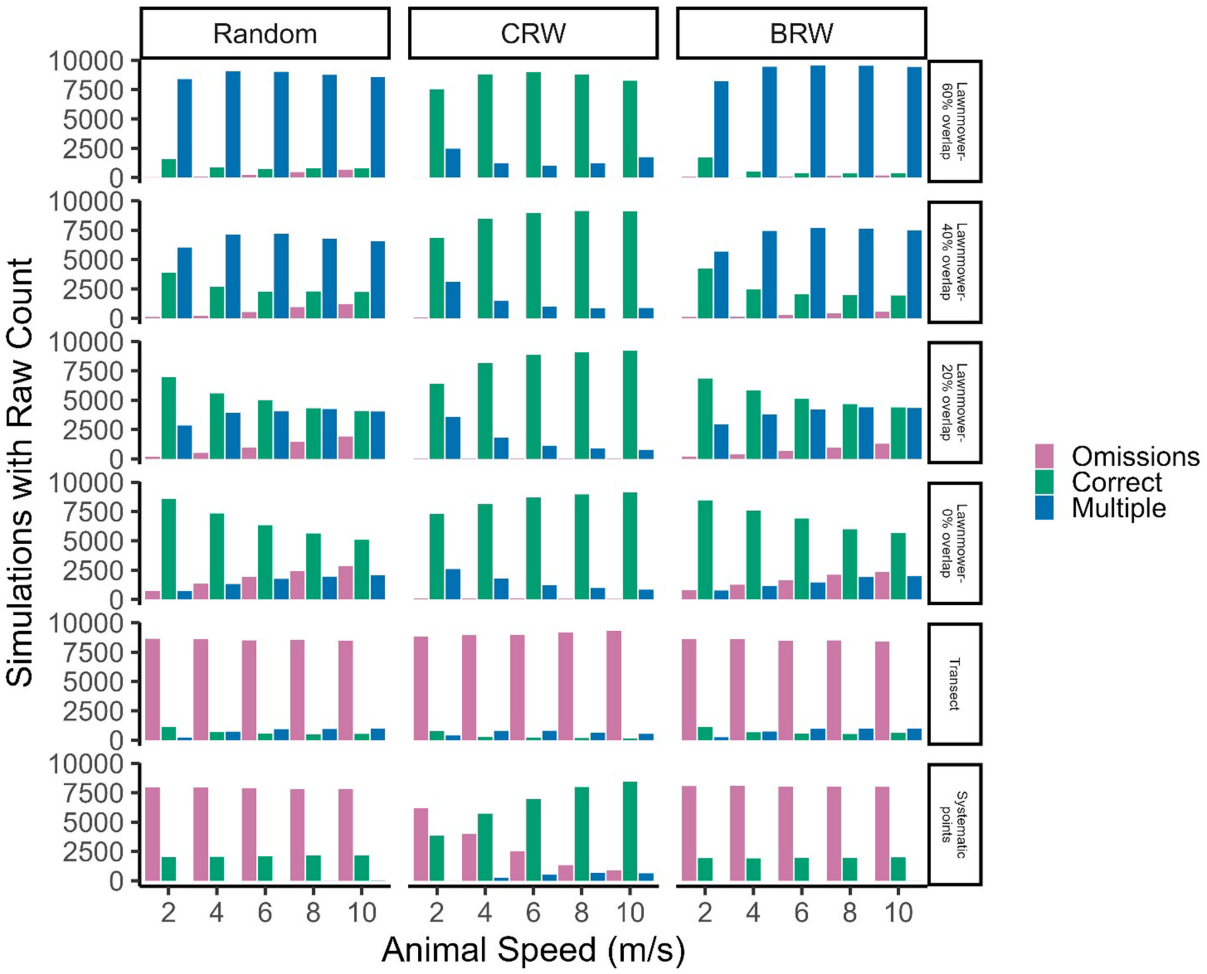
Total number of simulations from an agent‐based model that either returned the correct number of animals (one animal; Correct), omitted the animal (Omissions), or had multiple counts (Multiple) among various drone flight patterns, animal movement speeds, and animal movement patterns (BRW, biased random walk; CRW, correlated random walk; Random, random walk).

We simulated six drone flight patterns, which increased along a spectrum of spatial independence among images and included the following commonly used flight patterns: (1) a lawnmower pattern with 60% image overlap (Figure 1a), (2) a lawnmower pattern with 40% image overlap (Figure 1b), (3) a lawnmower pattern with 20% image overlap (Figure 1c), (4) a lawnmower pattern with 0% image overlap where images touched (Figure 1d), (5) a randomized belt transect (Figure 1e), and (6) systematic points (Figure 1f). To ensure the assumption that the animal was 100% available and detectable during the simulated survey, the landscape dimensions were slightly revised for the lawnmower patterns with 20, 40, and 60% image overlap to ensure complete coverage by the drone imagery. The lawnmower patterns with 20% and 40% image overlap covered a 242,064 m^2^ (492 × 492 m) landscape; whereas for 60% overlap, the landscape size was adjusted to 219,024 m^2^ (468 × 468 m). For the lawnmower pattern with 0% image overlap, transect, and systematic point flight patterns, the landscape size was fixed at 230,400 m^2^ (480 × 480 m).

Transect surveys included one horizontal belt transect with a length of 384 m (80% of the total landscape length) and a width of 60 m (image width; Figure 1e). Image captures from transects were programmed to have 60% frontal overlap, capturing imagery of 10% of the total landscape. Transects were generated to include stochasticity among simulations by randomly selecting the initial x and y coordinates for each replicate in places that would allow the entire transect to be placed horizontally across the landscape. The systematic points flight pattern simulated 16 image captures evenly distributed across the landscape (Figure 1f), which amounts to the same number of images captured by the transect survey. However, since the systematic points flight pattern did not exhibit any image overlap it was able to capture 25% of the total landscape. The animal was counted when it was located inside the image viewing window. To account for approaches where multiple images would be stitched into an orthomosaic (Frazier & Singh, 2021), an animal was not counted in an image if it had not moved more than 4 m from its previous location as the animal would have remained within the same grid cell. Previous studies describing “ghost” animal issues (Brack et al., 2018; Lenzi et al., 2023) do not detail how far animals moved when creating discrepancies, but in our case movements greater than 4 m were assumed to be large enough to cause issues with post‐processing software within the simulations.

### 2.3 Animal movement

To best quantify error rates, only one animal was simulated within the landscape so that counts >1 indicated multiple counting, whereas 0 or average counts <1 were associated with animal omission. By using one animal on the landscape, we were able to track all animal movements, isolate variables of interest, and address our study objectives in a relatively simplistic environment. The use of a single animal on this 230,400 m^2^ landscape was equivalent to a density of 4.3 animals/km^2^, which is like natural densities of many mammalian and large raptor populations (Kittle et al., 2017; Laurent et al., 2021; Roseberry & Woolf, 1998). Thus, our simulations apply to low density and solitary animal species.

For each survey type, the animal was first positioned randomly on the landscape. Initial validation simulations had no movement, mimicking a stationary animal for the entire survey duration as a control to compare to other simulation scenarios that subsampled the landscape. A moving animal was then simulated with one of three different movement patterns: (1) random walk, (2) correlated random walk, and (3) biased random walk. Walks were created by sampling an exponential step length distribution and varying turning angle distributions (see Appendix S1; Duchesne et al., 2015). For each walk type, simulations were run with average animal velocities representing a spectrum of natural terrestrial animal speeds (2, 4, 6, 8,10 m/s), as animal taxa differ substantially in various locomotion behaviors that affect speed (walking, running, etc.). To maintain standardized comparisons within the study purpose for drone surveys, the simulated animal was designed to only move within the closed landscape (i.e., no immigration or emigration) and was always available for detection within the viewing window of the drone (i.e., no occlusion). Count outputs also assumed that perception and detection probability during image review was perfect. If an animal reached the border of the landscape, depending on its programmed movement type, it was randomly reflected in a new direction and continued its programmed movements within the simulated landscape area until the drone survey was complete.

### 2.4 Simulations

A total of 90 scenarios were simulated with each combination of drone flight pattern (*n* = 6), animal movement pattern (*n* = 3), and animal speed (*n* = 5) iterated 10,000 times, resulting in a total of 900,000 simulations. For each simulation, the number of times the animal was captured within the image taken by the drone was recorded and the mean and standard deviation (SD) of the raw counts were reported for model replicates to compare various combinations of our variables. Accuracy of the survey counts was based on the deviation from the true value (i.e., one animal; Hone, 2008). We also report the percentage of simulations that returned the correct number of animals (*n* = 1), omitted the animal, or had multiple counts among scenarios. We compare subsampled landscape (transect and systematic point) counts to control scenario counts using a randomly placed, stationary animal on the landscape and report differences in mean and SD of the raw counts. To further visualize the differentiation of raw count error among animal walk, animal speed, and drone flight pattern, we conducted a regression tree analysis. We used the Classification and Regression Tree method (De'ath & Fabricius, 2000; Lewis, 2000) with raw count as the response variable and animal walk and speed, as well as drone flight pattern as predictor variables. We report variable importance values of each predictor as measures of effect size. A full description of the simulations, following the ODD protocol (Overview, Design concepts, and Details) for agent‐based models (Grimm et al., 2020), is provided in Appendix S1.

## 3 RESULTS

Flight pattern, animal movement pattern, and animal speed all affected the count bias. The regression tree analysis revealed that the effect of drone flight patterns were most influential for predicting the raw count of the survey (Figure 2). Variable importance values by predictor, ranked from greatest to least, were flight pattern (78), animal walk (17), and speed (6). Thus, the effect of flight pattern was over 4.5 times more than animal walk type, which was almost three times more than animal speed. With one animal on the landscape, the mean and standard deviation of animal counts ranged from 0.2 ± 0.7 to 3.2 ± 2.7 animals among flight patterns, from 1.1 ± 1.1 to 1.6 ± 2.1 animals among movement patterns, and 1.2 ± 1.2 to 1.5 ± 2.0 animals among animal speeds. Although flight pattern was the most influential variable determining accurate animal counts in drone surveys, combinations of various animal movement patterns and speeds also resulted in more accurate counts of the simulated animal within various flight patterns (Figures 3 and 4).

For flight patterns, the lawnmower pattern with 0% overlap was the least biased of all animal movement types and speeds (1.1 ± 0.6 animals, Figure 3) with comparatively high accuracy (73.2% of simulations with correct counts; Figure 4). The next most accurate flight pattern was the lawnmower pattern with 20% overlap (63% of simulations with correct counts) followed by the lawnmower with 40% and 60% overlap (45.7% and 33.6% of simulations with correct counts, respectively; Figure 4). Counts increased overall with lawnmower overlap percentage, averaging 1.4 ± 0.9, 1.9 ± 1.4, and 3.2 ± 2.7 animals for 20%, 40%, and 60% overlap, respectively (Figure 3). The transect and systematic point flight patterns were the most likely to omit the animal in the drone survey (0.2 ± 0.7 and 0.4 ± 0.5 animals, respectively; Figure 3). The transect flight pattern very rarely returned an accurate animal count across movements and speeds (Figure 3) and mostly omitted (87.1%) the animal, as did the systematic points (63.1%; Figure 4). As the transect flight pattern captured 10% of the landscape, it should have captured the animal in 10% of our simulations; however, the average count for a moving animal was 0.2 ± 0.7, indicating that animal movement influenced survey counts, especially when compared to the average of 0.1 ± 0.3 for the stationary animal transect count. In contrast, the systematic points flight pattern, with images covering 25% of the landscape, had a greater average count of 0.4 ± 0.5 mobile animals, compared with 0.25 ± 0.4 stationary animals.

Animal counts were most accurate for the correlated random walk (1.1 ± 1.1 animals) among drone flight patterns for almost all animal speeds (Figure 3). Generally, the random and biased animal walks resulted in overestimated animal counts (1.6 ± 2.1 and 1.6 ± 1.9 animals, respectively), particularly when overlap increased for lawnmower patterns from 20% to 60%, (Figure 3). The correlated random walking animal resulted in the least number of multiple counts (12.0%), with 36.0% and 37.7% of simulations having multiple counts for the random and biased random walking animal, respectively (Figure 4). Animal movement resulted in the omission of the animal in 20.3% (correlated random walk), 32.1% (biased random walk), and 33.7% (random) of simulations (Figure 4).

Varying the speed of the animal exhibited one clear trend among variables; increasing animal speed increased the variation around counts (i.e., lowered precision) for most flight patterns and animal walks (Figure 3). The lawnmower pattern with 60% overlap and, to a lesser extent the 40% overlap, tended to overestimate animal counts, with average counts and variability nearly doubling, as animal speed increased from 2 to 10 m/s during random and biased random walking (Figure 3). In contrast, increasing animal speed tended to decrease multiple counts for the correlated random walk (Figure 4). Animal speed also influenced the number of correct counts in some cases, with the percentage of correct survey counts decreasing for the 0% (66.0% and 69%) and 20% (51.8% and 53.7%) overlap for the random and biased walks, respectively, but increasing for the correlated random walk for those flight patterns (84.4% and 83.5%, respectively; Figure 4). The number of correct survey counts also increased with animal speed for systematic points when the animal had a correlated random walk (38.3% correct at 2 m/s to 84.6% correct at 10 m/s; Figure 4).

## 4 DISCUSSION

This research represents the first study investigating the interactions of multiple drone flight patterns and animal movement behaviors in a systematic and quantitative approach. We demonstrate that drone flight patterns can greatly influence animal count accuracy, from 4 to 13 times more than animal walk or speed, even over relatively small areas (herein ~22–24 ha). Our results also provide support for the use of a rarely considered drone flight pattern (a lawnmower pattern with 0% image overlap) for animal monitoring. While lawnmower patterns with large overlaps allow for the development of image mosaics for landscape mapping (Frazier & Singh, 2021), we found that these flight patterns increasingly lead to overestimated counts of mobile animals as percent overlap increased as predicted, even when accounting for mosaicking. Subsampling the landscape with a belt transect underestimated counts of the moving animal as predicted compared to a stationary animal. However, subsampling with systematic points was more accurate when the animal was moving compared with stationary, contrary to predictions. Increases in animal persistence and speed often did not result in overcounting the moving animal, as we predicted. Some directional persistence (random correlated walk) in the moving compared to the stationary animal resulted in more accurate counts than no (random walk) or greater directional persistence (biased walk). Increasing animal speed tended to decrease precision overall, but the results depended on flight pattern and walk type. Our results have important and often overlooked implications for drone surveys compared with more commonly applied practices.

Easily programmed drone lawnmower pattern surveys typically use 60%–80% overlapping imagery (Aubert et al., 2021; Lyons et al., 2019), but our results indicate this may have major implications for multiple‐count concerns during drone surveys if the animal of interest is mobile. While it is acknowledged that animals move during surveys (Brack et al., 2018), many drone surveys assume animals are stationary (Sudholz et al., 2022) and create a mosaic image to more easily count animals and understand distributions (De Kock et al., 2021; Ezat et al., 2018) without quantifying the effect of animal movement on counting accuracy. A few field drone studies have attempted to address animal movement issues post data collection (Linchant et al., 2018) and with manual image searches for clones, partial, or blurred animals after mosaicking (Barbedo & Vieira Koenigkan, 2018; Lenzi et al., 2023). Another approach reviews individual overlapping images, comparing animal shapes, sizes, and positions to reduce the number of multiple counted animals (Cleguer et al., 2021; Sudholz et al., 2022; Witczuk et al., 2018). This additional post‐processing of imagery can be helpful, but uncertainty remains in their effectiveness considering most animals are unmarked or indistinguishable from other individuals. These image reviews are also very labor intensive, time‐consuming, and do not address animal omissions due to their movements (Brack et al., 2018). Automated image classification approaches are being developed (Chabot et al., 2022; Dujon et al., 2021; Gonzalez et al., 2016; Krishnan et al., 2023), but the development of accurate algorithms for aerial animal imagery is still in its infancy and has many challenges to overcome (Corcoran, Winsen, et al., 2021; Sudholz et al., 2022). Addressing the issue of multiple counting during data collection, as opposed to during post‐processing could reduce the likelihood of the multiple‐count problem. Incorporating lawnmower patterns with minimal image overlap may be key, as noted in the increase in count accuracy of our simulations as overlap percentage decreased.

Subsampling the landscape or spreading sampling intervals has been suggested as a means to avoid issues of multiple counts of the same animal (Witczuk et al., 2018). However, we found that animal movement can still influence counts of an individual in these scenarios. The average count and variation of the transect flight pattern both doubled when the animal was moving, as opposed to when the animal was stationary on the landscape. Similarly, for the systematic points flight pattern, the average count increased 1.5 times, with a slight increase in count variation as well. However, we also found that during the systematic points flight (Figure 1f), an animal moving with directional persistence resulted in a large percentage of accurate surveys, which was consistent with other drone flight patterns. Thus, surveys at systematic points for animals behaving this way may be accurate and would result in less imagery for post‐processing, sequentially leading to additional time savings during data preparation and image evaluation. Ultimately, subsampling the landscape, compared to a full census, will require correction of counts (Buckland et al., 2001), but as we have shown, these corrections should vary depending on if the animals of interest are anticipated to be mobile during the survey period.

Our simulations confirm that animal movement patterns and speeds influence whether an animal is correctly counted in drone imagery. The random and biased random walking animal movement patterns often resulted in overestimates from multiple counts of an animal traveling back into the path of the drone after its initial “capture.” An increase in the animal speed lowered the precision for most flight patterns and animal walks, with an exception for the correlated walking animal. Therefore, researchers need to consider animal movement behaviors to avoid count bias and consequential incorrect management prescriptions (Guerrasio et al., 2022). Overall, our results emphasize that knowledge of animal movement patterns can help identify the optimal survey periods and drone flight patterns to minimize sampling error. To minimize count error, one might survey using a systematic points flight pattern during crepuscular periods when certain species, such as white‐tailed deer, are most active (Kammermeyer & Marchinton, 1977). Or depending on the research question, 0% overlap lawnmower pattern surveys during other times of day or year when individuals are more stationary, such as when juveniles have not yet dispersed from natal areas, may also be appropriate.

Even on our simplified landscape, we observed large amounts of bias among animal counts during scenario simulations with one mobile animal. While our assumptions of 100% availability and detectability are highly unlikely in real‐world applications (Gilbert et al., 2021), for example, due to visual obstructions above the animals or the ability of the animal to dive underwater or move under cover (Brunton et al., 2020; Hodgson et al., 2017), this assumption allowed us to simplify our scenarios and better understand how flight patterns and animal movements may create counting errors. Typically, surveyors are concerned with omission rates associated with conventional animal survey methods (i.e., occupied aircraft and ground surveys) due to detectability issues, and there are means of addressing some of these problems (Brack, Kindel, de Oliveira, & Lahoz‐Monfort, 2023; Hamilton et al., 2018; Samuel et al., 1992; Steinhorst & Samuel, 1989). For example, the inclusion of detection probabilities in statistical models has greatly improved our ability to estimate animal populations (Corcoran, Denman, & Hamilton, 2021; Martin et al., 2012), and incorporating detection probabilities into drone‐based estimates would be a helpful advancement (Brack, Kindel, de Oliveira, & Lahoz‐Monfort, 2023; Hodgson et al., 2017, 2023). It is also notable that false positives (i.e., multiple counts) are less frequent during ground‐based and occupied aircraft surveys, something that researchers using drones need to carefully consider moving forward (Brack et al., 2018).

We acknowledge other trade‐offs must be considered for drone surveys such as balancing battery life and line‐of‐sight limitations during survey planning (Baxter & Hamilton, 2018; Linchant et al., 2015). Hence, trade‐offs between the area sampled and survey accuracy may need to be considered for larger sampling areas. There may also be potential for increased accuracy with alternative flight patterns that we did not consider. For example, sea turtle density estimates were calculated using a modified strip–transect approach with 35%–45% frontal overlap and sequential images used in counts to reduce multiple counting potential (Brack, Kindel, Berto, et al., 2023; Sykora‐Bodie et al., 2017), and point count drone surveys with 360‐degree rotations were found to be a promising approach for mesocarnivore abundance estimates (Bushaw et al., 2019). In either case, the method was not thoroughly vetted for accuracy, although this could be explored in future work. Barbedo and Vieira Koenigkan (2018) suggest flying multiple drones in formation to collect accurate counts, acknowledging that animals could otherwise move between survey efforts. However, they also note that this would greatly increase survey cost and that formation flights have many technical challenges. In any case, researchers should not assume mosaics composed of overlapped images can be used for both vegetation mapping and animal surveying simultaneously. Instead, careful thought is needed for drone flight patterns with objectives related to animal monitoring.

## 5 CONCLUSIONS

As the use of drones in animal monitoring continues to grow, consideration of how these survey platforms can be appropriately incorporated into animal survey techniques is vital. Based on our results, when using a drone to survey areas similar to our simulations (~22–24 ha), we recommend that, researchers interested in animal counts should consider a lawnmower flight pattern with 0% overlap as an alternative to other more easily programmed, overlapping patterns. We also recommend that animal life history knowledge be incorporated in survey design, aligning with stages and times of day when animals may exhibit more sedentary or more directional movements. This will allow for the most accurate counts as well as maximize overall ground coverage area when accounting for limited battery capabilities (Linchant et al., 2015). The simulated approach we utilized also allows for robust inference to investigate a myriad of animal behaviors and population processes that can be broadly applied across many taxa and provide guidance of drone applications in a variety of wildlife management applications. Although our scope in this study was limited to solitary, low‐density animals, future efforts with our agent‐based modeling approach can help assess the influence of animal density, distributions, and detection probabilities to better simulate real‐world environments.

## AUTHOR CONTRIBUTIONS

**Emma A. Schultz:** Conceptualization (equal); data curation (lead); formal analysis (lead); methodology (equal); writing – original draft (lead); writing – review and editing (equal). **Natasha Ellison‐Neary:** Conceptualization (equal); data curation (equal); formal analysis (equal); methodology (equal); software (equal); validation (equal); writing – original draft (equal); writing – review and editing (equal). **Melanie R. Boudreau:** Conceptualization (equal); methodology (equal); writing – review and editing (equal). **Garrett M. Street:** Conceptualization (equal); methodology (equal); project administration (equal); writing – review and editing (equal). **Landon R. Jones:** Conceptualization (equal); methodology (equal); writing – review and editing (equal). **Kristine O. Evans:** Conceptualization (equal); methodology (equal); writing – review and editing (equal). **Raymond B. Iglay:** Conceptualization (equal); funding acquisition (lead); methodology (equal); project administration (equal); writing – review and editing (equal).

## FUNDING INFORMATION

This work was funded by the FWRC and MAFES of Mississippi State University and United States Department of Agriculture Agricultural Research Service Non‐Assistance Cooperative Agreement no. 58–0200–0‐002 via the Graduate Summer Research Experience in High‐Performance Computing and Agriculture fellowship program in the Geosystems Research Institute at Mississippi State University.

## CONFLICT OF INTEREST STATEMENT

The authors have no conflict of interest.

## Supporting information


Appendix S1.

Appendix S2.

## Data Availability

Data can be found in Appendix S2.
