# Supplementary material for: On the move: Influence of animal movements on count error during drone surveys
Source: Ecol Evol. 2024 Sep 29;14(10):e70287. doi: 10.1002/ece3.70287 (PMC11439628; doi:10.1002/ece3.70287)
Supplement: Supplementary file 1 — Appendix S1. [file ECE3-14-e70287-s002.docx]

**Appendix.**

**Supporting Information – Methods: ODD description of drone and animal simulation model**

The model description follows the ODD (Overview, Design concepts, Details) protocol for describing agent-based models (Grimm *et al.*, 2020). Our agent-based model was developed in Python version 3.9 (<https://www.python.org/>).

**1. Purpose and Patterns**

The purpose of this simulation is to compare the efficiency of counts of various drone (Unoccupied Aircraft Systems or UAS) flight patterns when surveying a landscape with one mobile animal exhibiting multiple animal movement patterns at various movement speeds. The results of this simulation assess the importance of drone flight patterns when investigating animal counts. The main objective of this model is to provide robust inference for drone survey applications in remote wildlife detection.

**2. Entities, state variables, and scales**

The model consists of one animal and one drone on a simulated open landscape. The speed and viewing window for image acquisition of the drone agent are fixed, and movement paths depend on six drone flight scenarios (i.e., patterns). The animal agent moves according to three movement types and five speed levels. The landscape is a homogenous, square area where the drone and animal move unimpeded. Each cell on the landscape (x, y coordinate) represents a 4 x 4 m area, with the total area surveyed dependent on the flight pattern scenario as described herein.

**3. Process overview and scheduling**

Each simulation is initialized with the drone starting in the northwest corner of the landscape (i.e., top left corner of square). This closely represents the real-world scenario of drone flight deployment; an investigator is likely to launch the drone on or outside the edge of the interest area to avoid disturbance to animals and for logistical convenience. Timesteps are equivalent to 0.4 s intervals based on the simulated drone flight speed of 10 m/s. At each time step, the drone moves according to the simulated flight pattern. The image capture rate is also based on the drone flight pattern simulation. At each time step, a mobile animal moves within the landscape with the direction and distance of movement based on the modeled walk and speed. The simulation ends when the drone has reached the furthest eastern extent of the landscape that it can survey within its programmed rules. At the end of the simulation, an output of the total animal count for that run was exported. Each simulation was run 10,000 times for each combination of drone flight patterns and animal movements, totaling 900,000 model runs.

**4. Design concepts**

*Basic principles:*

The objective of the simulation is to quantify error associated with monitoring a mobile animal with a mobile drone platform. Animal monitoring efforts utilizing drones have increased within the last decade (Elmore *et al.*, 2023) and continue to rise with further technological advancements. It is logical that animal movements will influence counting accuracy of these surveys through double counting or omission of individuals (Brack, Kindel and Oliveira, 2018). However, to date, there has been no research to quantify the error of various drone flight patterns or determine which flight patterns may be the most effective for monitoring mobile animals.

*Emergence:* The emergent properties of the model are the movement patterns of the animal agent during various drone flight patterns.

*Adaptation:* Agent behaviors are not adaptive in the model.

*Objectives:* Agents do not seek any direct objectives in the model.

*Learning:* Adaptive behaviors that include learning do not exist in the model.

*Prediction:* Agents do not make any predictions in our model.

*Sensing:* Agents do not exhibit sensing in the model.

*Interaction:* Two agents (drone and one animal) are modeled at the same time within one simulation. The drone interacts with the animal by taking images of the animal as the drone passes over it, but no behavioral response of the animal to the drone is modeled.

*Stochasticity:* The starting location of the animal is stochastic for each simulation run at a random location on the landscape. The transect drone flight pattern has stochasticity within the horizontal starting location, which determines its full horizontal length in the landscape.

*Collectives:* No collectives are simulated in our model.

*Observation:* The simulation output is the count of animals located within the drone viewing window during image capture over the duration of the survey. Resulting counts are then compared to the known number of animals on the landscape (n = 1).

**5. Initialization**

The start location of the drone exhibiting lawnmower patterns is in the northwest corner of the landscape. The transect flight pattern starts at a randomly selected y-coordinate on the landscape and is 384 m in length with horizontal stochasticity for starting location. Values for x and y are randomly selected as the start coordinate for the animal on the landscape at the beginning of each simulation.

**6. Input data**

Input parameters for the animal movements are distributions of step lengths and turn angles. Step length is the distance an animal moved between consecutive locations (e.g. Murray, 1988; Levey *et al.*, 2005) and varied based on modeled animal walk behaviors. Turn angles also varied by animal walk type.

**7. Submodels**

*Drone platform and movements*

The drone parameters and viewing window are representative of a 20-megapixel camera with a focal length of 6.8 mm with a field of view of approximately 67 degrees flown at 61.0 m (200 ft) above ground level, representing a ground sample distance of 1.28 cm. The total viewing window simulated is 60 x 60 m. The drone is simulated at 10 m/s flight speed with each timestep of the model equal to 0.4 s.

*Drone survey scenarios*

The six drone flight patterns modeled include: (1) a lawnmower pattern with 60% image overlap, (2) a lawnmower pattern with 40% image overlap, (3) a lawnmower pattern with 20% image overlap, (4) a lawnmower pattern with 0% image overlap where images touched, (5) a randomized linear transect, and (6) systematic points. For the lawnmower pattern with no image overlap, transect, and systematic points flight patterns, the landscape was 230,400 m^2^ (480 x 480 m). The lawnmower 0% overlap pattern simulated image capture with only image edges touching. The transect flight pattern started at randomly selected x and y coordinates on the landscape and was 384 m in length with horizontal stochasticity for the starting location. Images were captured with 60% frontal overlap across the transect. The systematic points flight pattern captured 16 images evenly across the landscape. The lawnmower patterns with 20% and 40% image overlap covered a 242,064 m^2^ (492 x 492 m) landscape. The landscape size was adjusted to 219,024 m^2^ (468 x 468 m) for the lawnmower pattern with 60% image overlap. Landscape size adjustments ensured complete coverage of the area by the drone imagery to keep the assumption that the animal was 100% available and detectable on the landscape during “census” approaches. The lawnmower pattern with 20%, 40%, and 60% overlap was programmed to capture images with 20%, 40%, and 60% frontal and side overlap, respectively.

*Animal Movement*

Animal speeds of 2, 4, 6, 8, and 10 m/s were modeled, changing the position of the animal at each timestep to capture a range of movements applicable to a wide range of animal species of various body sizes and movement capabilities from slow walking to running. The animal agent was programed to exhibit three animal movement patterns: random walk, correlated random walk, and biased random walk. Walks were created by varying step length and turning angle distributions (Duchesne, Fortin and Rivest, 2015). For a random walking animal, the animal was initially placed on the landscape at a random location. An exponential function was randomly sampled, with the mean for each step length based on the programmed speed of the animal. Then a turning angle between 0 and 360 degrees was sampled from a uniform distribution. Each new animal position was generated using the previous position and the sampled step length and turning angle.

For a correlated random walking animal, a Von mises distribution was used to sample turning angles with the corresponding parameters set to mu = 0 and kappa = 1. The current heading of the animal was determined with the inverse square tangent of the coordinate. Each new animal position was then generated using the same process as the random walk.

For a biased random walking animal, the animal was also initially placed at a random location on the landscape and a den was randomly placed no further than 20 cells (80 m) away from the animal, resulting in commonly observed patterns of animal space use such as home ranging behavior (Codling, Plank and Benhamou, 2008). The current heading of the animal was determined by subtracting the x and y coordinates of the den location from the position of the animal and then taking the inverse square tangent of the coordinate. A Von mises distribution was used to sample a turning angle where mu = current animal heading and kappa = 0.25, therefore biasing the animal towards the den. Each new animal position was generated in the same way as the random walk. For all walks, if the animal was on the edge of the landscape, a new random turn angle was selected to keep the animal inside the landscape parameters.

*Model validation, image capture, and output*

A stationary animal was randomly placed on the landscape for each combination of drone flight pattern approaches for verification of the model and to compare against animal movement simulations. An animal was counted when it was located within the drone viewing window at the time image capture was simulated. The model output accounts for mosaicking of the simulated image captures. When an animal moves less than the distance of a cell (4 m) between subsequent images, the animal is only counted once assuming that a human would recognize that individual as the same animal in subsequent images. The output of the simulation is the total number of animals counted by the drone across the landscape.

**References**

Brack, I.V., Kindel, A. and Oliveira, L.F.B. (2018) ‘Detection errors in wildlife abundance estimates from Unmanned Aerial Systems (UAS) surveys: Synthesis, solutions, and challenges’, *Methods in Ecology and Evolution*, 9(8), pp. 1864–1873. Available at: https://doi.org/10.1111/2041-210X.13026.

Codling, E.A., Plank, M.J. and Benhamou, S. (2008) ‘Random walk models in biology’, *Journal of The Royal Society Interface*, 5, pp. 813–834. Available at: https://doi.org/10.1098/RSIF.2008.0014.

Duchesne, T., Fortin, D. and Rivest, L.-P. (2015) ‘Equivalence between Step Selection Functions and Biased Correlated Random Walks for Statistical Inference on Animal Movement’, *PLOS ONE*, 10(4), p. e0122947. Available at: https://doi.org/10.1371/JOURNAL.PONE.0122947.

Elmore, J.A. *et al.* (2023) ‘Evidence on the efficacy of small unoccupied aircraft systems (UAS) as a survey tool for North American terrestrial, vertebrate animals: a systematic map’, *Environmental Evidence*, 12.

Grimm, V. *et al.* (2020) ‘The ODD Protocol for Describing Agent-Based and Other Simulation Models: A Second Update to Improve Clarity, Replication, and Structural Realism’, *Journal of Artificial Societies and Social Simulation*, 23(2). Available at: https://doi.org/10.18564/JASSS.4259.

Levey, D.J. *et al.* (2005) ‘Effects of landscape corridors on seed dispersal by birds’, *Science*, 309(5731), pp. 146–148. Available at: https://doi.org/10.1126/science.1111479.

Murray, K.G. (1988) ‘Avian seed dispersal of three neotropical gap-dependent plants’, *Ecological Monographs*, 58(4), pp. 271–298. Available at: https://doi.org/10.2307/1942541.
